# A framework for analyzing both linkage and association: an analysis of Genetic Analysis Workshop 16 simulated data

**DOI:** 10.1186/1753-6561-3-s7-s98

**Published:** 2009-12-15

**Authors:** E Warwick Daw, Jevon Plunkett, Mary Feitosa, Xiaoyi Gao, Andrew Van Brunt, Duanduan Ma, Jacek Czajkowski, Michael A Province, Ingrid Borecki

**Affiliations:** 1 Division of Statistical Genomics, Washington University School of Medicine, 4444 Forest Park Boulevard, Campus Box 8506, St. Louis, Missouri 63108 USA

## Abstract

We examine a Bayesian Markov-chain Monte Carlo framework for simultaneous segregation and linkage analysis in the simulated single-nucleotide polymorphism data provided for Genetic Analysis Workshop 16. We conducted linkage only, linkage and association, and association only tests under this framework. We also compared these results with variance-component linkage analysis and regression analyses. The results indicate that the method shows some promise, but finding genes that have very small (<0.1%) contributions to trait variance may require additional sources of information. All methods examined fared poorly for the smallest in the simulated "polygene" range (*h*^2 ^of 0.0015 to 0.0002).

## Background

Both linkage analysis and association analysis provide useful, but slightly different, forms of information in identifying genetic contributions to complex traits. Frequently, either one or the other type of analysis is employed, which can result in information being overlooked: linkage does not account for the association frequently seen between disease alleles and nearby markers, while association (on family data) may account for family structure, but does not make use of the location information that the meiotic events provide. When both are used, the analyses are typically done under different frameworks. Here, we consider an integrated analysis of both linkage and association under an oligogenic model [1] and compare it with a linkage method and an association method.

## Data

For Genetic Analysis Workshop (GAW) 16, a simulated data set was provided based on the Framingham Heart Study. Multiple replicates of the simulation were provided to help assess methods and we used several of these replicates for this purpose. This simulation used ~550,000 single-nucleotide polymorphisms (SNPs) (GeneChip^® ^Human Mapping 500 k Array Set and the 50 k Human Gene Focused Panel) from actual Framingham data, also provided to GAW16. Traits were simulated on to these SNPs by selecting several SNPs as oligogenes (*h*^2 ^of 0.01 to 0.001) and 1000 SNPs as polygenes (*h*^2 ^of 0.0015 to 0.0002) for each trait. Some of the "polygenes" have effects as large as those of the "oligogenes." As in real data, the boundary between the two is fuzzy. The polygenes were selected more randomly, although some were selected as clusters. We focused our analysis on chromosome 11 and simulated low-density lipoprotein (LDL) and high-density lipoprotein (HDL) at the first visit. For linkage, we selected two subsets of 1-cM spaced markers with a simple algorithm that traversed the chromosome at 1-cM distances given two starting points. Linkage information is captured almost completely by markers at this density. Thus, adding additional markers only provides a marginal increase in information, while increasing the computational burden [2]. We selected two subsets to control for both potential tight associations between markers and trait genes and for the potential effects of undetected typing error. We examined our ability to identify both oligogenes and polygenes, including clusters of polygenes, and in particular, if there is any benefit to conducting linkage and association analysis in a common framework.

## Methods

We used Markov-chain Monte Carlo (MCMC) methods to produce Bayesian statistics for segregation, linkage, and association. These methods are implemented in the computer program Loki [1]. Covariate effects are also estimated, and the trait model is given by:

where *μ *is the "reference" trait value, *X *is the incidence matrix for covariate effects, *β *is the vector of covariate effects, *Q*_*i *_is the incidence matrix for the effects of quantitative trait locus (QTL) *i*, *α*_*i *_is the vector of effects for QTL *i*, *e *is the normally distributed residual effect, *k *is the number of QTLs currently estimated (*k *≥ 0), *S*_*j *_is the incidence matrix for the effects of SNP *j*, *γ*_*j *_is the vector of effects for SNP *j*, and *l *is the number of SNPs being tested for association in the analysis run. The MCMC process samples *μ*, *β*, *α*_*i*_, *γ*_*j*_, *i*, and *e *as well as parameters such as unobserved marker genotypes and QTL genetic position. All of these parameters are sampled from the space of model values consistent with the data observed. Values are sampled proportional to their posterior probability. After the number of sampling iterations is sufficiently large, the sampled values provide an estimate of the posterior probability distribution over the parameter space. The difference between this and previous applications of the method is in the number of SNPs we test. Previously, we have included select candidate genes as genetic covariates. Here we will test all the SNPs available on chromosome 11 for association with this method to examine how the method scales up to genome-wide association testing.

Initially, LDL was analyzed with the two 1-cM SNP subsets for linkage only (*l *= 0). Subsequently, single SNPs and sets of SNPs were added to test for association. When testing SNPs for association, we considered 1) *z*-scores for each of the elements of *γ*_*j *_being non-zero, which assessed the strength of the association, and 2) whether adding SNP *j *reduced any linkage signal found in the region, which indicated that SNP *j *was associated with the mutation causing the linkage signal. If a single SNP in a region produced the linkage signal, including it for association testing should eliminate the linkage peak, by moving the effect from the segregation term () to the association term (). Genetic effects in the segregation term were tested for linage to the included markers. Genetic effects in the association term were those of typed genes and thus not reflected in the linkage signal. Even when not testing for linkage, including the segregation term could improve association testing as a result of allowing explicit modeling of the effects of loci other than the one tested. To examine this hypothesis, we conducted analysis runs with only one SNP for all ~26,000 SNPs on chromosome 11.

To evaluate evidence for linkage, we used Bayesian "*L*-scores" estimated over 1-cM bins along the chromosomes. An *L*-score is simply the posterior probability divided by the prior probability. In the absence of any data, the posterior probability should be equal to the prior probability. Thus, a *L*-score of 1 indicated that the data contained no information for or against linkage, while a *L*-score >1 indicated evidence for linkage.

For comparison, we also conducted linkage analysis with the computer program SOLAR, and association analysis with a family-based test implemented with PROC MIXED in the computer program SAS [3]. In addition, we computed *r*^2 ^for age- and sex-adjusted HDL in 2-cM intervals, sliding 1 cM at a time across chromosome 11. For this regression analysis, we used PROC REG in SAS with forward selection and an inclusion threshold of 0.1 for including a SNP in the regression. These *r*^2 ^values give an indication of the strength of association in each replicate, whether due to causative loci or random chance. In particular, they provide an indicator of the predictive information present for association present in the data.

## Results

**Figure 1:**
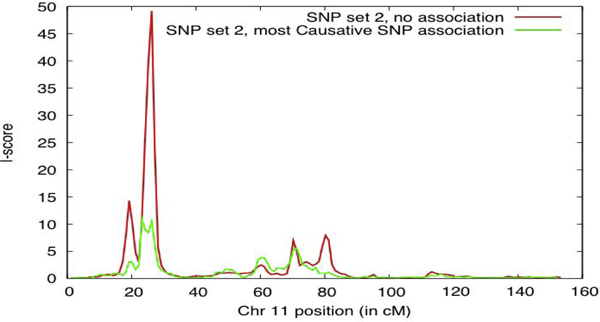
**Associated SNPs account for linkage peaks**. L-scores for MCMC oligogenic linkage analysis of LDL with (green) and without (red) simultaneous association testing at most causative SNPs. Including the causative SNPs reduces the linkage signal in most regions.

First, we compared the MCMC linkage *L*-scores with and without simultaneous association testing at most causative SNPs (Figure 1). Some SNPs could not be placed on the linkage map and so were excluded. The linkage scores were reduced, as expected, and 19 out of the 62 SNPs had association |*z*-scores| > 2. All of these SNPs were part of the simulation model, so ideally, we would like to see more |*z*-scores| > 2. However, the small effects of some of these SNPs (with *h*^2 ^< 0.001) led to insufficient power. These results indicate that this method can be useful in candidate gene studies.

**Figure 2:**
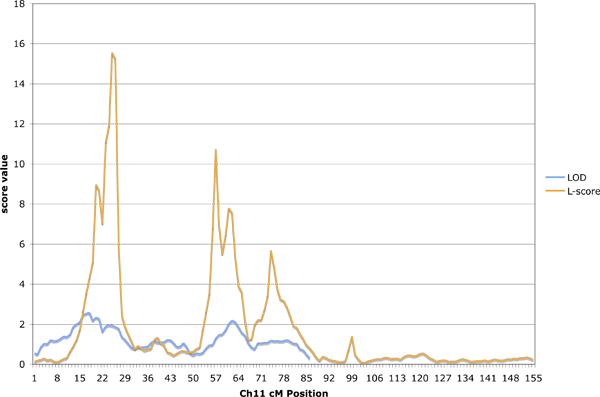
**Agreement between methods within replicate**. Comparison of linkage results for simulated LDL at Visit 1, adjusted for age and sex, for L-scores from oligogenic simultaneous segregation and linkage analysis (without association testing) and for LOD from variance-components methods. Vertical scales are not comparable, but note agreement of peak locations

**Figure 3:**
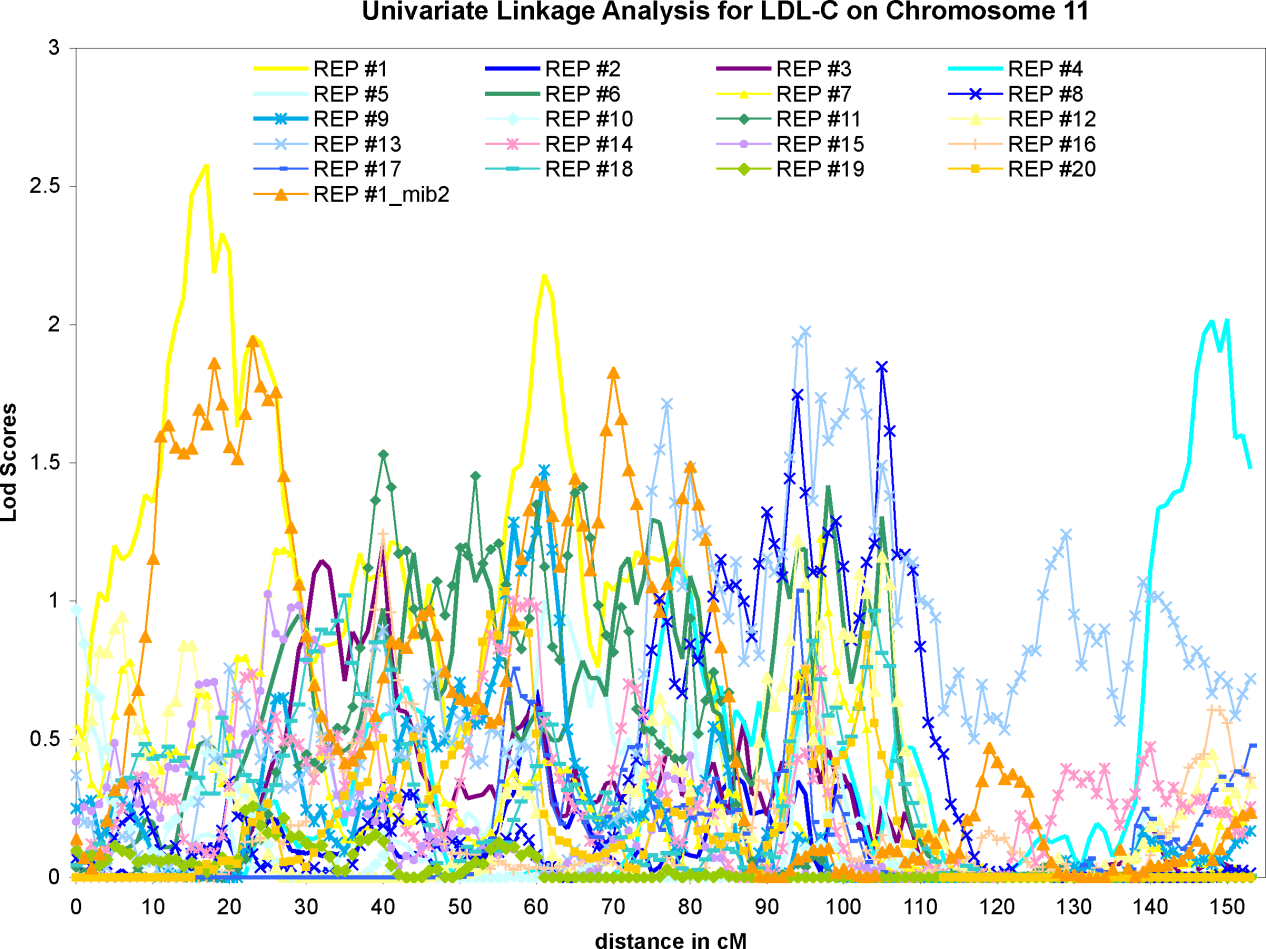
**Different signals in different replicates**. LDL SOLAR linkage results. While several regions exhibited LOD > 1 in multiple replicates, no region did so in all replicates.

In comparison of the variance-component linkage results and MCMC oligogenic linkage, we found that within a simulated replicate for this data set, the two methods produce similar results (Figure 2). However, between replicates, the locations in which the signals were found differed, sometimes markedly (Figure 3). This difference holds for both linkage methods, so we included only the LOD plots, which are more familiar to most. We note that the two different subsets of 1-cM distant SNPs produced concordant linkage signals within the same replicate (see yellow and gold lines in Figure 3). The discordance between replicates likely reflects that although there were many signals on each chromosome, the power to map these signals, most with *h*^2 ^< 0.001, was marginal and so different replicates may have had different signal strength at different locations.

**Figure 4:**
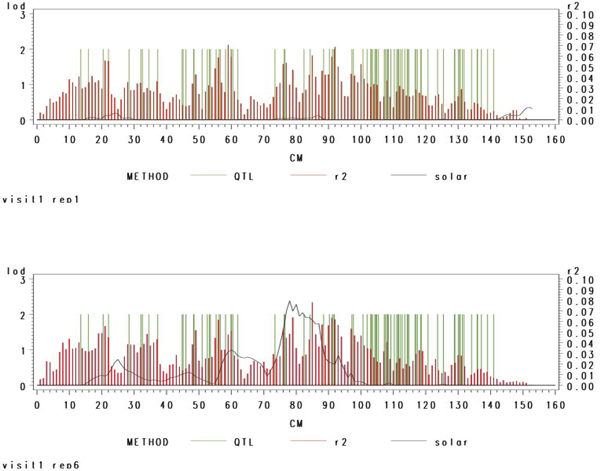
**Linkage and Association vs. gene locations**. HDL Visit 1 adjusted for age and sex analyzed SOLAR and regression analyses in 2-cM wide windows (moving in 1-cM increments). In Replicate 1, no evidence for linkage was found. In Replicate 2, a LOD score > 2 at ~80 cM was found.

To further explore between-replicate variation, we computed *r*^2 ^explained within 2-cM intervals. In Figure 4, we show these values (red), the LOD scores (black), and the locations of the causative SNPs (green bars) for two replicates. There is variation in both the LOD and *r*^2 ^between replicates and neither seems to reflect the concentration of SNPs around 110 cM. These results suggest that both linkage and association tests alone might fare poorly within gene clusters with a multitude of mutations with each *h*^2 ^< 0.001.

Finally, in single-SNP association tests under the MCMC oligogenic model, ~1,700 out of ~26,000 SNPs had |*z*-scores| > 2. This is slightly greater than the 5% expected under the null hypothesis. With many simulated "causative" SNPs, nearly all *z*-scores could be argued to be "near" a causative SNP, and thus there was no empirical null in this data. These single-SNP tests were computationally intensive, and probably not practical on a genome-wide scale with current technology. There were some positive signals here, so an alternate methodology may facilitate these tests with dense SNPs. As currently implemented, these methods are best suited to combined linkage and candidate gene association studies.

## Discussion

Oligogenic analysis of combined linkage and candidate gene association appears to work for genes with an *h*^2 ^on the order of 0.01. We had issues detecting some of the "polygenes" in this simulation (*h*^2 ^of 0.0015 to 0.0002), but all methods appear to lack power to detect the smallest of these "polygene" effects. At the margin of such effects, it is important to extract as much information from the data as possible and our motivation here was to examine the benefits of combining both linkage and association.

Here, we saw that there was some benefit to examining both types of information simultaneously. However, the lack of power to detect the very smallest "polygenes" is cause for concern. If the genes in this simulation are not unrealistically smaller than those that exist in real traits, these results suggest that many true positives could be due to random reinforcing of true signals, and replication will be difficult. It could be that the very smallest effects in the simulation may be undetectable. The results of computed *r*^2 ^explained in regression computed over 2-cM intervals are discouraging because of the poor correspondence between gene locations and *r*^2 ^peaks. However, there does appear to be room for improvement.

The methods used here, in their current state, are very useful for testing of candidate gene associations. Fully incorporating genome-wide association study data will require algorithmic improvements. In particular, while there were many *z*-scores with an absolute value >2, they do not survive multiple testing corrections at the genome-wide association study level. Incorporating additional sources of information, such as that on gene networks, might help with this issue. While our success here was more limited than we hoped, incorporating information from multiple sources in a single framework may help detect marginal genetic signals.

## List of abbreviations used

GAW: Genetic Analysis Workshop; HDL: High-density lipoprotein; LDL: Low-density lipoprotein; MCMC: Markov-chain Monte Carlo; QTL: Quantitative trait locus; SNP: Single-nucleotide polymorphism

## Competing interests

The authors declare that they have no competing interests.

## Authors' contributions

EWD conceived and coordinated the study, participated in its design, carried out the MCMC analyses, and drafted the manuscript. JP performed the variance-component linkage analysis and plotted the results. MF participated in the design and helped with the variance-component linkage. XG participated in the design. DM carried out the association analysis. JC helped prepare the data and design algorithms for marker selection. MAP participated in the design. IB participated in the design.
